# Electrospun Cadmium Selenide Nanoparticles-Loaded Cellulose Acetate Fibers for Solar Thermal Application

**DOI:** 10.3390/nano10071329

**Published:** 2020-07-08

**Authors:** Nicole Angel, S. N. Vijayaraghavan, Feng Yan, Lingyan Kong

**Affiliations:** 1 Department of Mechanical Engineering, The University of Alabama, Tuscaloosa, AL 35487, USA; nmangel@crimson.ua.edu; 2 Department of Metallurgical and Materials Engineering, The University of Alabama, Tuscaloosa, AL 35487, USA; vsankaranarayanannair@crimson.ua.edu; 3 Department of Human Nutrition and Hospitality Management, The University of Alabama, Tuscaloosa, AL 35487, USA

**Keywords:** photoactive nanoparticles, cadmium selenide, cellulose acetate, electrospun fibers, solar thermal

## Abstract

Solar thermal techniques provide a promising method for the direct conversion of solar energy to thermal energy for applications, such as water desalination. To effectively realize the optimal potential of solar thermal conversion, it is desirable to construct an assembly with localized heating. Specifically, photoactive semiconducting nanoparticles, when utilized as independent light absorbers, have successfully demonstrated the ability to increase solar vapor efficiency. Additionally, bio-based fibers have shown low thermal conductive photocorrosion. In this work, cellulose acetate (CA) fibers were loaded with cadmium selenide (CdSe) nanoparticles to be employed for solar thermal conversion and then subsequently evaluated for both their resulting morphology and conversion potential and efficiency. Electrospinning was employed to fabricate the CdSe-loaded CA fibers by adjusting the CA/CdSe ratio for increased solar conversion efficiency. The microstructural and chemical composition of the CdSe-loaded CA fibers were characterized. Additionally, the optical sunlight absorption performance was evaluated, and it was demonstrated that the CdSe nanoparticles-loaded CA fibers have the potential to significantly improve solar energy absorption. The photothermal conversion under 1 sun (100 mW/cm^2^) demonstrated that the CdSe nanoparticles could increase the temperature up to 43 °C. The CdSe-loaded CA fibers were shown as a feasible and promising hybrid material for achieving efficient solar thermal conversion.

## 1. Introduction

Currently, clean freshwater scarcity is a significant issue that is closely linked with social and economic development [1,2,3]. Already, billions of people worldwide lack access to safe drinking water, and millions die annually due to diseases relating to water-borne illnesses [2,4]. Some proposed solutions for addressing water scarcity work at the expense of aggravating present energy problems, while others suggest the implementation of large-scale infrastructure [1,4]. Therefore, developing a method to address water scarcity in a clean, affordable, and sustainable manner is of increasing importance. Solar thermal techniques provide an affordable way to convert solar energy to thermal energy; for instance, solar vapor generation for water desalination has been demonstrated as a potential technique to provide a sustainable solution to water scarcity [1,3,5,6].

One of the main drawbacks of this technique is low solar thermal conversion efficiency [1]. Both the low solar absorption of the water as well as heat loss due to the use of conventional water heating systems have significantly contributed to this low efficiency [1]. Therefore, shifting from a bulk water heating system to a nanoscaled solar absorber system with localized heating capability, specifically through the implementation of photosensitive nanoparticles (NPs), has demonstrated a noticeable increase in conversion efficiency [1,5,6]. To form a localized heating structure, it is desirable to couple high thermal conductive, photoactive NPs with a low thermal conductive polymer matrix (such as a bio-based polymer). The thermal energy generated from the photoexcited carriers in the NPs become localized heat that has difficulty diffusing into the surrounding polymer substrate; thus, the localized heating of water is enabled [7]. This advantage of localized heating can overcome the remarkable heat dissipation that occurs during bulk water heating via conventional semiconductor abosrbers. Beneficially, this process does not require the total surrounding liquid volume to reach its boiling point for successful vapor generation [6]. 

In this study, we investigate the implementation of electrospun cellulose acetate (CA) fibers carrying photosensitive cadmium selenide (CdSe) NPs as a nanoscaled solar conversion device for solar thermal conversion. CdSe, a Group II-VI compound semiconductor, exhibits extraordinary electronic and optoelectronic properties [8,9]. CdSe displays optimal sunlight absorption with a bandgap of 1.74 eV [10] and a high thermal conductivity (−0.53 W/cmK) [11]. Cellulose acetate is a low-cost cellulose derivative produced via the acetylation of cellulose [12,13] with low thermal conductivity (−0.10 W/cmK) [14]. It was chosen as the fiber material in this study due to its advantageous mechanical properties, excellent fiber-forming ability, biodegradability, stability in water, and cost-effectiveness [13,15]. Electrospinning has become a popular method for the synthesis of micro- and nanofibrous materials due to the use of relatively simple manufacturing equipment, low spinning cost, variety of spinnable materials, and highly controllable processes [16,17]. Moreover, micro- to nanoscale fibers display a high surface-area-to-volume ratio, good flexibility, high porosity, and superior stiffness and tensile strength than when compared to other forms of the spun material [15]. Note that, for the seawater desalination application, the heavy toxic metal element, i.e., Cd, may diffuse into the water via photocorrosion and lead to water contamination. Here, we use the CdSe as a model semiconductor with a suitable sunlight absorption band edge to explore the potential to load into CA fibers through electrospinning technique. Other low-toxic and nontoxic semiconductor particles with desired light absorption capability, such as Sb_2_S_3_, Fe_2_O_3,_ and Fe_3_O_4_, will be explored in future studies. In particular, the diffusion behavior of the metal elements during the water desalination will also be investigated later. 

## 2. Materials and Methods

### 2.1. Materials

Cellulose acetate (MW ∼100,000 Da; acetyl content ~39.7 wt%) and acetone were purchased from VWR International (Radnor, PA, USA).

Based on the slow reaction between Cd^2+^ and Se^2−^ ions in an aqueous basic bath with pH > 10, the CdSe nanoparticles were synthesized using a chemical bath deposition process. Cadmium sulfate (CdSO_4_) and sodium selenosulfite (Na_2_SSeO_3_) were used as the sources for Cd^2+^ and Se^2−^, respectively. Na_2_SSeO_3_ was prepared by dissolving elemental Se in the form of fine powder in an aqueous solution of sodium sulfite heated to 60 °C. The solution was stirred well until the Se was completely dissolved. The pH of the solution was adjusted by adding excess NaOH. CdSe was formed in 2 h at a temperature of 70 °C. The obtained CdSe powder was washed using deionized water, centrifuged repeatedly, and subsequentially dried in a vacuum oven. 

Because CdSe is considered toxic to human cells, it was handled with the utmost care during this study [18]. CdSe toxicity has been mainly attributed to the release of Cd^2+^ ions from the CdSe to surrounding cells [18]. The method of exposure by which this diffusion occurs highly alters how significantly the affected cells react to CdSe [18]. Consequently, equipment in contact with CdSe was cleaned thoroughly, waste was properly disposed of, and the CdSe NPs were properly labeled and stored before and after use.

### 2.2. Methods

#### 2.2.1. Electrospinning

In this study, the CA concentration in the spinning dope was held constant at 12% (w/v). To prepare the spinning solutions, CA and CdSe were mixed in pure acetone via magnetic stirring at room temperature (20 °C) and then ultrasonicated (VWR International, Radnor, PA, USA). The spinning dope was then loaded into a 10-mL syringe (Becton, Dickinson and Company, Franklin Lakes, NJ, USA) with a 22-gauge blunt needle (Hamilton Company, Reno, NV, USA) as the spinneret.

The electrospinning setup was comprised of a high voltage generator (ES30P, Gamma High Voltage Research, Inc., Ormond Beach, FL, USA), a syringe pump (NE-300, New Era Pump Systems, Inc., Farmingdale, NY, USA), and a grounded aluminum foil as the collector. In the present study, the optimal spinning parameters to spin CA fibers were determined from our previous study [15]. Specifically, the spinning distance was held at 8 cm, the feed rate at 3 mL/h, and the voltage at 12 kV. Electrospinning was conducted in a fume hood at 20 °C without airflow. Airflow was removed due to the high evaporation rate of acetone. Eliminating airflow helped to slow the buildup of viscous fluid at the spinneret tip that could directly cause the destabilization of the spinning jet. Periodically, fiber formation can be halted because the spinneret can become clogged and the jet will no longer be considered continuous. Consequently, despite the lack of airflow in this study, the spinneret required constant cleaning during the spinning process to successfully fabricate fibers [19]. After the spinning process, the formed fibers, which had deposited on the aluminum foil directly beneath the spinneret tip, were collected and stored away from light and moisture for further analysis.

#### 2.2.2. Scanning Electron Microscopy (SEM)

The morphology of the electrospun fibers was studied using an Apreo field emission SEM (FE-SEM, Thermo Fisher Scientific, Waltham, MA, USA), equipped with energy-dispersive X-ray spectroscopy (EDS), at an accelerating voltage of 20 kV. Images were subsequently analyzed using ImageJ software (National Institute of Health, Bethesda, MD, USA). Three images were used for each fiber sample. Ten random segments on each image were taken and used to measure the fiber diameters. Through visual inspection, fiber morphology was evaluated as either good, fair, or poor [20]. Good fibers were defined as continuous, uniform, smooth, and defect-free. Fair fibers exhibited a fibrous shape with moderate defects. Poor fibers had significant defects, such as beads or sprayed particles. Notably, the CdSe NPs altered how smooth the fibers appeared, but were not considered to be fiber defects.

#### 2.2.3. Wide-Angle X-ray Diffraction (XRD)

The wide-angle X-ray diffraction (XRD) patterns of the fibers were obtained using a Philips X’Pert Materials Research Diffractometer (Malvern Panalytical, Westborough, MA, USA) operated at 45 kV under 40 mA Cu Kα radiation (λ = 0.15405 nm).

#### 2.2.4. Ultraviolet-Visible (UV-vis) Spectroscopy

The light absorbance of the fiber samples were measured using EnliTech QE measurement system (Kaohsiung City, Taiwan). The incident wavelength was swept from 300 to 1100 nm, and the absorbance of the fibers were recorded. The optical direct bandgap (Eg) of the fibers was determined by using Tauc plot relation to the plot (αhν)^1/r^ versus the energy of the photons, where *α* is the absorption coefficient of the materials, and *r* represents the nature of the transition of the charge carriers, and r = ½ for direct bandgap materials.

#### 2.2.5. Solar Energy Conversion

Using a compact infrared (IR) camera (FLIR C2, FLIR Systems, Inc., Wilsonville, OR, USA), the fibers were tested for solar vapor generation under 1 sun (100 mW/cm^2^) illumination. The thermal images were recorded after the nanofibers were exposed to the simulated sunlight for 30 min to stabilize in the air between heating and cooling. 

## 3. Results and Discussion

### 3.1. Electrospinnability and Fiber Morphology

The CA fibers were fabricated by electrospinning, a simple, scalable, and versatile technique for nanofiber production [21]. Given the potential applications of this study, the ability for our resulting solar vapor generator to be mass scaled is of utmost importance. The basic lab-scale electrospinning setup only requires a high voltage source, a syringe with a blunt needle tip (i.e. the spinneret), and a grounded collector [15,21]. High voltage is applied to the spinneret where the spinning dope is pumped via a syringe pump at a constant rate. Once the applied voltage reaches the critical point, a Taylor cone will form at the spinneret tip. A continuous jet flows from the Taylor cone to the grounded collector. During this process, the electric field causes the jet to stretch and elongate as the solvent evaporates. Fibers form on the grounded collector, e.g. the aluminum foil, as used in this study [15].

**Figure 1:**
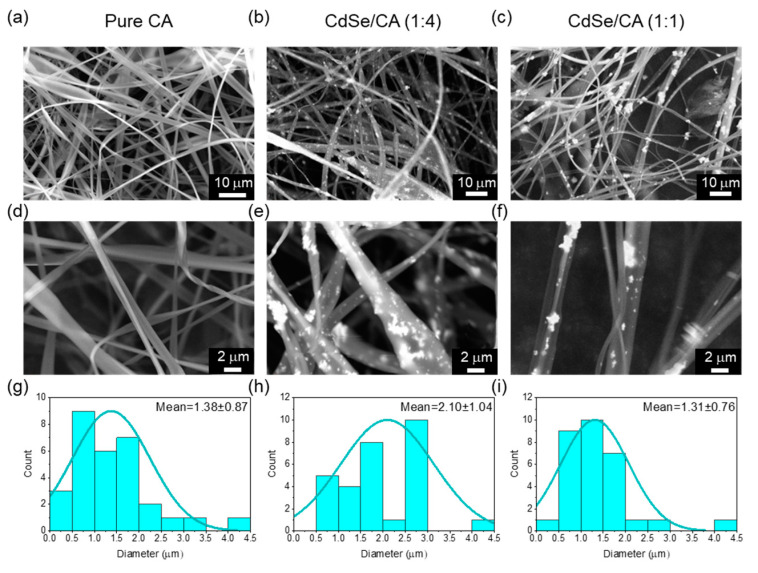
(**a**–**f**) Field emission scanning electron micrographs (FE-SEM) images and (**g**–**i**) fiber diameter distributions of CA, CdSe-CA (1:4), and CdSe-CA (1:1) fibers, respectively.

The CA fibers were loaded with varying CdSe concentrations (CdSe:CA ratio of 1:4 and 1:1, w/w) to analyze the successful uptake of CdSe into the CA fibers and to observe the resulting photosensitive properties of the fibers as provided by the CdSe NPs. The overall electrospinnability of the CdSe-CA compositions was analyzed through both visual inspection during spinning and the obtained fiber images (Figure 1). All three CdSe-CA dispersions were able to successfully produce fibers in a relatively short amount of time. The time needed for the collector to become completely covered in fibers was about the same for all three runs, suggesting that CdSe did not have a drastic effect on the electrospinnability of CA in the single solvent acetone. The overall quality of the electrospun fibers was also independent of the addition of CdSe. As seen in Figure 1, the CdSe-CA fibers remain continuous and smooth, discounting the CdSe NPs, and display approximately the same amount of beading as the pure CA fibers. These fibers were thus rated as good fibers, albeit their size was on the microscale rather than the nanoscale.

### 3.2. CdSe Incorporation

**Figure 2:**
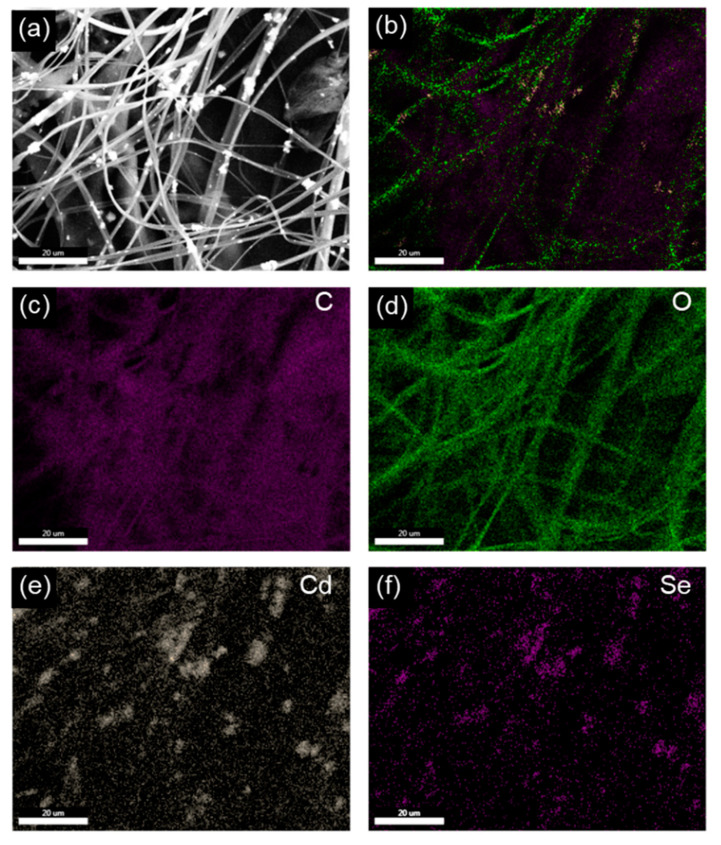
(**a**) FESEM, (**b**) overall elemental distribution, and (**c**–**f**) C, O, Cd, and Se elemental distribution of CdSe-CA (1:1) fibers obtained using energy dispersive X-ray spectrometry (EDS) mapping.

As shown in Figure 2, CdSe was successfully incorporated into the CA fibers. The white particles embedded in the fibers were confirmed to be comprised of elements Cd and Se, respectively (Figure 2c–f). As expected, CdSe-CA (1:1) fibers demonstrated a higher quantity of successful CdSe incorporation as compared with CdSe-CA (1:4) fibers. However, in both the (1:1) and (1:4) fibers, the CdSe particles were segregated in clusters rather than being evenly dispersed. This could be a result of the difficulty in achieving a homogeneous distribution and stable suspension of CdSe in the spinning dope. Additionally, during electrospinning, the CdSe particles may be charged and easily segregated together.

**Figure 3:**
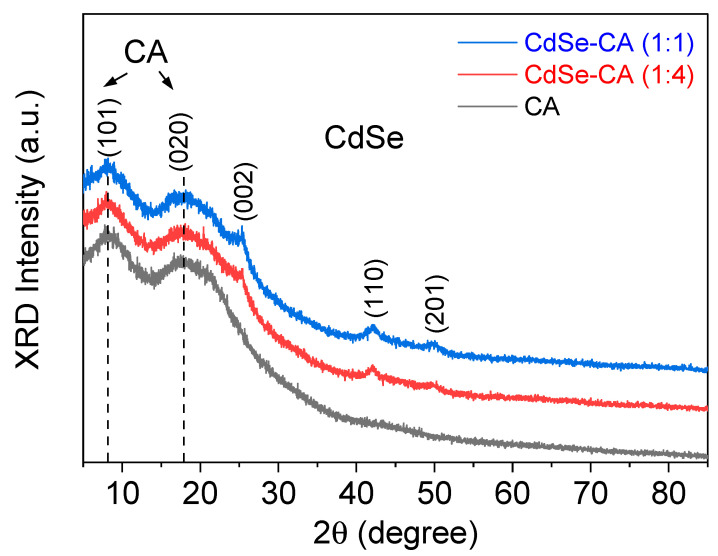
X-ray diffraction patterns of CA, CdSe-CA (1:4), and CdSe-CA (1:1) fibers.

The structure of the CdSe loaded CA fibers was characterized using X-ray diffraction (XRD), as shown in Figure 3. The XRD peaks of CdSe at 2θ = 25.3°, 42°, and 50° were observed in the CdSe-loaded fibers, whereas these peaks did not exist in the pattern of the pure CA fibers. The (002), (110), and (201) peaks of CdSe, corresponding to 2θ ~ 25°, 41°, and 50°, respectively, can be indexed to the wurtzite phase. Similar to most other biopolymers, CA is semi-crystalline yet largely amorphous. The diffraction peaks around 10° and 20°, corresponding to the (101) and (020) planes, respectively, confirm the semi-crystalline nature of CA [22]. When the CdSe/CA ratio increased, the intensity of the CdSe peaks increased.

### 3.3. Optical Properties

**Figure 4:**
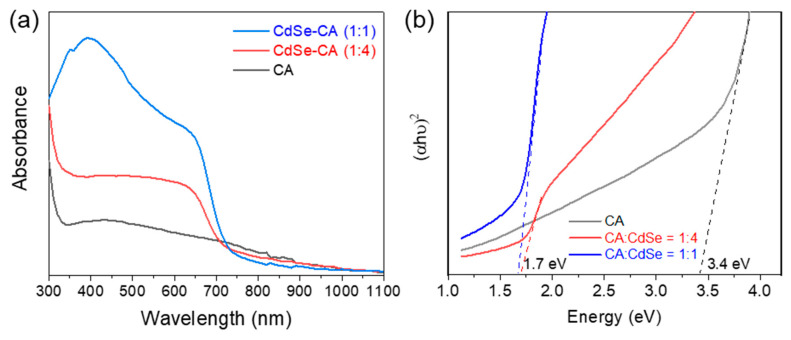
(**a**) UV-vis absorbance spectra (normalized at 1100 nm) and (**b**) Tauc plot of CA, CdSe-CA (1:4), and CdSe-CA (1:1) fibers.

As shown in Figure 4a, the absorbance of the CA fibers increased as the CdSe content increased, with the light absorption edge around 700 nm for the CdSe-loaded CA fibers. These fibers have superior sunlight absorption than pure CA fibers which indicates increased solar thermal conversion [23]. From the extrapolation of the Tauc plot, as shown in Figure 4b, CA was determined to have a wide bandgap of ~3.4 eV which corresponds to the ultraviolet light range in the solar spectrum. When CdSe was introduced into CA fibers with varying concentrations, the CdSe-CA (1:4) fibers were found to have a bandgap of ~1.7 eV. When more CdSe was incorporated into CA in the CdSe-CA (1:1) fibers, the bandgap remained constant. Note that pure CdSe is estimated to have a bandgap of 1.74 eV according to previous works [10]. The fact that the bandwidth of the CdSe-loaded fibers is close to that of pure CdSe demonstrates that loading CdSe into CA fibers can significantly increase the sunlight absorption of CA fibers. Therefore, CA fibers show great potential in their ability to act as a supporting matrix for CdSe NPs-based solar thermal conversion for improved localized heating.

### 3.4. Photothermal Conversion

**Figure 5:**
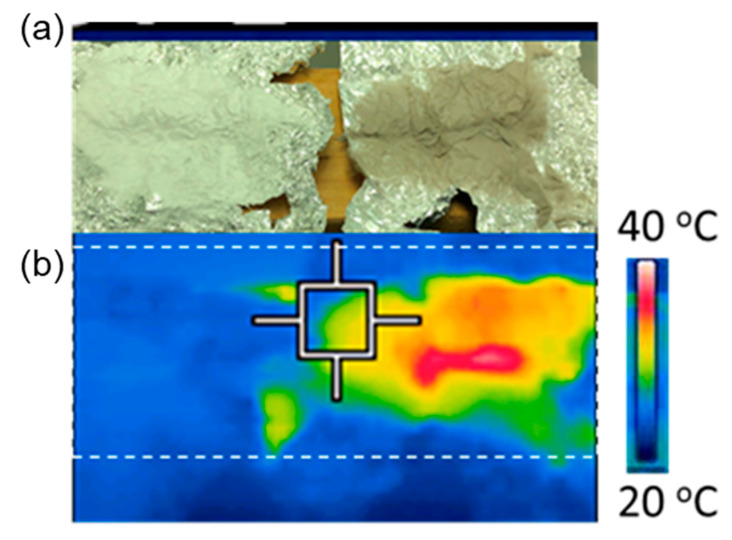
(**a**) Optical picture and (**b**) infrared thermal image of the CA and CA-CdSe fiber mats.

To investigate the solar thermal properties of the CA and CA-CdSe fibers, both optical and IR thermal images were captured (Figure 5). As seen in the optical image, the CdSe-CA (1:4) fibers were dark due to the light absorption by CdSe, while the pure CA fibers were white, which is in agreement with the UV-Vis absorbance test above. The maximum temperature in the CdSe-CA (1:4) fibers can be over 40 °C under the 1 sun (100 mW/cm^2^) illumination, while the pure CA fibers maintained a low temperature without photothermal conversion. This solar thermal conversion performance is similar to the carbonaceous membrane assisted by localized heating design [24]. It is suggested that the CA-CdSe fibers could effectively absorb sunlight and convert it into thermal energy.

## 4. Conclusions

In conclusion, the loading of highly thermal conductive CdSe NPs into CA fibers of low thermal conductivity was successfully demonstrated in this study. Overall, fiber morphology, including quality and diameter, was relatively independent of the addition of CdSe. The solar energy absorption of CdSe NPs were virtually unaffected while in the fibers. Specifically, the bandwidth of CdSe NPs remained the same while in the CA fibers, and the final assemblies were highly absorbent in the visible light spectrum. It was demonstrated that electrospinning can effectively introduce photosensitive NPs into the bio-based CA fibers. This low-cost and scalable technique can be used to facilitate water desalination through solar thermal evaporation with localized heating that has the potential to help address the current concerns regarding water scarcity encountered across the globe.
